# The effect of intranasal insulin on appetite and mood in women with and without obesity: an experimental medicine study

**DOI:** 10.1038/s41366-022-01115-1

**Published:** 2022-04-09

**Authors:** Elizabeth Schneider, Maartje S. Spetter, Elizabeth Martin, Elizabeth Sapey, Kay Por Yip, Konstantinos N. Manolopoulos, Abd A. Tahrani, Jason M. Thomas, Michelle Lee, Manfred Hallschmid, Pia Rotshtein, Colin T. Dourish, Suzanne Higgs

**Affiliations:** 1 School of Psychology, University of Birmingham, Edgbaston, Birmingham, B15 2TT UK; 2 Centre for Human Brain Health, University of Birmingham, Birmingham, UK; 3 Birmingham Acute Care Research Group, University of Birmingham, Birmingham, UK; 4 University of Birmingham Institute of Inflammation and Ageing, Birmingham, UK; 5 Institute of Metabolism and Systems Research (IMSR), College of Medical and Dental Sciences, University of Birmingham, Birmingham, UK; 6 Centre for Endocrinology, Diabetes and Metabolism, Birmingham Health Partners, Birmingham, UK; 7 School of Psychology, Aston University, Birmingham, UK; 8 Department of Psychology, Swansea University, Swansea, UK; 9 Department of Medical Psychology and Behavioural Neurobiology, University of Tübingen, Tübingen, Germany; 10 German Center for Diabetes Research (DZD), München-Neuherberg, Germany; 11 Institute for Diabetes Research and Metabolic Diseases of the Helmholtz Center Munich at the University of Tübingen (IDM), Tübingen, Germany; 12 P1vital Ltd., Wallingford, UK; 13 P1vital Products Ltd, Wallingford, UK

**Keywords:** Feeding behaviour, Translational research

## Abstract

**Background/Objectives:**

Intranasal (IN) administration of insulin decreases appetite in humans, but the underlying mechanisms are unclear, and it is unknown whether IN insulin affects the food intake of women with obesity.

**Subjects/Methods:**

In a double-blind, placebo-controlled, crossover design, participants (35 lean women and 17 women with obesity) were randomized to receive 160 IU/1.6 mL of IN insulin or placebo in a counterbalanced order in the post prandial state. The effects of IN insulin on cookie intake, appetite, mood, food reward, cognition and neural activity were assessed.

**Results:**

IN insulin in the post prandial state reduced cookie intake, appetite and food reward relative to placebo and these effects were more pronounced for women with obesity compared with lean women. IN insulin also improved mood in women with obesity. In both BMI groups, IN insulin increased neural activity in the insula when viewing food pictures. IN insulin did not affect cognitive function.

**Conclusions:**

These results suggest that IN insulin decreases palatable food intake when satiated by reducing food reward and that women with obesity may be more sensitive to this effect than lean women. Further investigation of the therapeutic potential of IN insulin for weight management in women with obesity is warranted.

Insulin is well known to act in the periphery to control blood glucose levels and promote nutrient storage. However, because the amount of insulin released is in direct proportion to the amount of body fat, and because it is able to cross the blood-brain barrier to enter the brain, insulin also acts as a central nervous system signal about levels of body fat, and simulation of brain insulin receptors is associated with reduced appetite and weight loss [1, 2]. Targeting of these central effects of insulin in the absence of the peripheral metabolic effects can be achieved by using the intranasal (IN) route of administration of insulin in humans [3]. Acute administration IN insulin reduces food intake in healthy adults [4–7] and longer term administration suppresses hunger and decreases body fat content [8] suggesting potential for weight management. However, not all studies have found effects of IN insulin on appetite and body weight [9–11]. To address these inconsistencies it is important to investigate the underlying mechanisms.

IN insulin may influence brain reward and homeostatic mechanisms to reduce food intake. In humans, IN insulin decreases functional Magnetic Resonance Imaging (fMRI) signals in the hypothalamus, a key centre of homeostatic control [12] that modulates the dopaminergic reward system [13–15]. IN insulin reduced intake of cookies in women when administered after a satiating lunch, but not in the fasted state, indicating that IN insulin might enhance satiation [16]. IN insulin also decreased cookie palatability [16], suggesting that a potential underlying mechanism is a reduction in the reward value of food.

IN insulin modulates neural activity in brain regions associated with higher order cognitive processes including the hippocampus and prefrontal cortex [17–19] and improves cognitive performance, particularly hippocampal-related memory [4, 9, 10]. Memory has been shown to influence appetite [20], for example, enhancing memory of recently eaten food reduces later intake [21]. Therefore, IN insulin may reduce food intake by enhancing memory. However, there has been no comprehensive assessment of the effects of IN insulin on food intake, homeostatic, reward, cognitive and neural processes.

Another important gap in understanding the actions of IN insulin on food intake is how they vary as a function of body fat mass and insulin resistance. Individuals with obesity may be less sensitive to IN insulin and have cerebral insulin resistance. For example, in contrast to men with obesity, lean individuals report a reduction in wanting of sweet foods after IN insulin [22]. Furthermore, effects of IN insulin on fMRI signals elicited by food stimuli are weaker or absent in individuals with type 2 diabetes or obesity [13, 15, 18, 22]. The effects of IN insulin on food intake in women with obesity have not yet been examined.

This is the first multimodal study to investigate the acute effects of IN insulin on palatable food intake and hunger in satiated women with and without obesity. To assess underlying mechanisms, we included behavioural measures of homeostatic, reward, and cognitive processes and used fMRI to assess effects of IN insulin on neural activity. We also examined the effects of IN insulin on emotional responses, as mood can impact weight management interventions. We predicted that IN insulin would reduce food intake, pleasantness of food and neural activity in brain reward areas. In addition, we explored whether IN insulin enhances cognitive performance, and predicted that any effects may be less pronounced in women with obesity.

## Research design and methods

### Participants

Fifty-four participants were recruited, but one lean and one participant with obesity withdrew during the first test day due to discomfort with having blood taken and experiencing a migraine respectively. The resulting sample consisted of thirty-five lean women and seventeen women with obesity. Assuming a small to medium effect size with 80% power, we aimed to recruit 35 participants in each BMI group but difficulties in recruitment resulted in a smaller sample size for women with obesity. Therefore, effect sizes are presented with all statistical outcomes. Participants were invited to the University of Birmingham Wellcome Trust Clinical Research Facility to take part if they met the eligibility criteria and passed a screening session. Participants were recruited via posters and social media platforms and were compensated with £100. Participants provided written informed consent and the research was conducted according to Good Clinical Practice. The study was approved by the Research Ethics Service (RG_17-102) and was pre-registered on clinicaltrials.gov as NCT03632681.

### Design

In a double-blind, placebo-controlled, crossover design, participants were randomized to receive 160 IU/1.6 mL of IN insulin (Actrapid; Novo Nordisk, Bagsværd, Denmark) or 160 IU/1.6 mL of placebo in a counterbalanced order. The dose was based on previous studies that reported significant effects of insulin on food intake [4, 6, 16, 23]. Placebo and insulin were identical in appearance and odour. Placebo consisted of water, 2.7 mg/ml m-cresol/mL, and 16 mg/mL glycerol (prepared by Guy’s and St Thomas’ NHS Foundation Trust’s Pharmacy Manufacturing Unit, London, UK). Placebo and insulin sprays were prepared by a pharmacist so that the study personnel and participants were unaware of group allocation. All participants took part in two sessions on two separate days at least 7 days apart. All procedures including insulin administration were overseen by medically qualified staff.

### Screening session

Height and weight were measured and the participant had a medical check. Questionnaires were completed to check eligibility and characterize the sample: The Structured Clinical Interview for DSM-5, Clinical Version (SCID-CV) [24], The Beck Depression Inventory – II (BDI-II) [25], The Dutch Eating Behaviour Questionnaire (DEBQ) [26], and The Power of Food Scale (PFS) [27] (See Supplementary Table 1 for eligibility criteria).

### Eating measures

Participants ate a lunch of cheese sandwiches (~588 kcal per sandwich). The portion size of which was adjusted for each participant to comprise 40% of daily energy requirements. The average portion served to each participant was 787.51 kcal (SD = ± 133.56, minimum = 565.57 kcal, maximum = 1143.56 kcal). Participants were asked to consume all of the lunch provided. Lunch was consumed prior to dosing because IN insulin has been reported to decrease consumption in women only in a postprandial state [16]. 140 min post-dosing participants were offered palatable Maryland® Chocolate Chip Cookies (along with a glass of water). Palatable cookies were chosen to allow us to examine food reward related responses. The cookies were broken up to disguise portion size (80 g) and served on a Sussex Ingestion Pattern Monitor (SIPM), which consists of a balance placed underneath the surface of a table covered by a placemat [28]. The balance was connected to a laptop that recorded the weight of the plate and alerted the participant to complete VAS ratings of hunger, fullness, and pleasantness each time 10 g of cookies was consumed (the participant was not aware of how much had been consumed). When 60 g of cookies were consumed, participants were provided with a fresh bowl of 80 g and could continue until they wished to stop. Eating rate was calculated as grams eaten/time spent eating.

### Appetite / Mood measures

#### Visual analogue scales (VAS)

Participants rated how they felt at that moment in relation to 14 sensations (alertness, drowsiness, happiness, hunger, fullness, desire to eat, thirst, disgust, anxiety, sadness, withdrawn, lightheaded, nausea, faint) by placing a vertical mark through a 10 cm horizontal line with left and right anchors indicating the extremes of each sensation (‘completely absent’ to ‘most I could imagine’) [28, 29].

#### Positive and negative affect schedule (PANAS)

The PANAS is a 20-item scale that is a reliable and valid measure of positive and negative affect [30].

### Cognitive tasks

#### Delay discounting

To assess ability to delay reward gratification participants completed a monetary discounting task that included nine delays. Participants saw the question ‘Which would you prefer?’, with two choices: £xx now or £xx after a delay (varying from one day to one year) and selected the preferred option. They also completed a food version in which they chose between a smaller amount of food now and a larger amount later, for example ‘Which would you prefer?’ with the options ‘one bite of chocolate now or a bar of chocolate in a month?’. Data are expressed as area under the curve [31, 32].

#### Verbal paired associates (VPA)

To assess hippocampal memory participants memorized 60 associated word pairs that were presented for 2 seconds on a computer screen. Immediately after (and then again after an hour delay) they received a cue word and responded aloud with the target word [33].

#### N-Back

To assess working memory blue circles were presented on a white 3×3 grid for 500 ms. Participants indicated if the circle was in the same position or a different position as it was two (2-back) and three trials back (3-back). Accuracy and reaction times (RT) for correct responses by stimuli (2 and 3-back) were recorded [34].

#### P1vital^®^ oxford emotional test battery

##### Emotional categorisation task (ECAT)

(see [35]; www.p1vital.com) Sixty positive and negative adjectives were presented for 500 ms. Participants indicated whether they would like or dislike to be described as such. RT by valence and accuracy (selection of positive adjectives for self-reference/rejecting negative adjectives for self-reference) were measured.

##### Emotional recall task (EREC)

Participants were given four minutes to recall as many words from the ECAT task as they could within 4-mins. Accuracy and commission errors by valence were analyzed.

##### Emotional recognition memory task (EMEM)

Participants were presented with 60 personality descriptor words, along with 60 matching novel distractor words and indicated whether the word had been presented during the ECAT. Percentage accuracy, RT for correct responses and commission errors were analyzed by valence.

### fMRI picture rating task

Participants viewed food and non-food pictures (36 from each category and visually matched) [36]. The food pictures varied in fat and sugar content (high fat, high sugar; high fat low sugar; low fat high sugar and low fat, low sugar). Items were rated on appeal using a scale from 1 (not at all) to 5 (very much). Each picture was presented for 1500 ms, followed by a fixation cross (500–1500 ms). At the end of the test day, participants were asked to recall as many of the pictures as possible and to record these responses on paper. Accuracy (percentage correct recalled) by category was analyzed.

### Acquisition, processing and analysis of fMRI data

Data were collected using a 3-Tesla Phillips Achieva MRI scanner with a 32-channel head coil at the Birmingham University Imaging Centre. Functional images during the picture task (3 × 185 volumes) were acquired with a single-shot echo-planar imaging (EPI) sequence: repetition time (TR = 2400 ms), adjusted flip angle (77°), echo time (TE = 30 ms), and 43 transverse slices (voxel size = 3 × 3 × 2 mm^3^). A gradient echo field map was also acquired (echo time (short) = 9.2 ms, echo time (long = 11.5 ms), flip angle = 90°). The T_1_-weighted anatomical scan was acquired with a TR/TE of 7.4/3.5 ms, a flip angle of 7°, FOV of 256 × 256 × 176 mm, 176 sagittal slices, and a voxel size of 1×1×1 mm^3^. Data were analysed using SPM12 standard procedures [37]. For each subject a model contained 4 regressors: four food stimulus categories and four matched non-food stimulus categories resulting in two contrast images: food versus matched non-food pictures. Six motion parameters were included. Contrast images from subject level were entered into a second level within-subjects ANOVA (insulin versus placebo).

### Blood insulin and glucose

Four blood samples (4 mL) were collected via an intravenous catheter inserted into an antecubital vein of the forearm of the participant’s choice at baseline, 5, 135 and 155 min post-IN insulin administration for the determination of haemoglobin (HbA1C; mmol/mol) on test day one and insulin and glucose on both test days. A small sample of blood collected from the intravenous catheter was tested at each timepoint to check for hypoglycaemia as a safety precaution. Blood samples were kept on ice or stored at −80 ˚C until centrifuged at 1500 × *g* for 15 min. At the end of the test day, capillary blood glucose was measured to ensure euglycaemia prior to the participant’s departure.

### Procedure

Participants completed baseline VAS and PANAS, had a medical check and blood draw. They then consumed lunch and afterwards completed a set of the VAS/PANAS and self-administered the IN insulin by inhaling 0.1 mL of insulin or placebo in a single puff. There were eight puffs per nostril giving a total of 1.6 mL and 30-second intervals between puffs. 5 min post-IN insulin administration, there was a blood draw and VAS/PANAS completed.

**Fig. 1:**
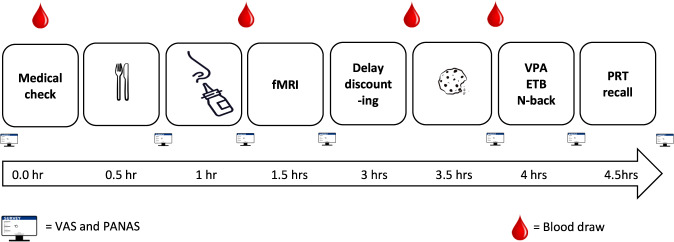
Procedure. Schematic overview of the test day procedure.

Participants then underwent the fMRI scan for 1.5 h during which they completed an inhibition task (results not reported here) and the picture rating task. Participants then completed the DD task, VAS/PANAS and had another blood draw, after which they consumed chocolate cookies. The final blood draw was taken and another set of VAS/PANAS completed. The participants then completed the immediate recall phase of the VPA, the N-back and ETB followed by VAS/PANAS. The delayed recall phase of the VPA was then completed. Next, the participants had 5 min to recall the images that were presented in the scanner and then completed a final set of VAS/PANAS. See Fig. 1.

### Analysis

Performance based exclusion criteria were determined prior to analysis. Outlying data points defined as <200 ms and ≥6000 ms for RT and outside 3*interquartile range of the lower and upper grand mean for other performance measures were removed. Participants scoring at below chance performance on the EMEM and N-back tasks were removed from the respective analysis. Six participants (3 lean and 3 participants with obesity) were missing more than 75% of blood draws and were removed from blood analyses. Eight participants were excluded from fMRI analysis due to excessive movement (*n* = 6), no second scan (*n* = 1) and missing data (*n* = 1). The final sample size for fMRI analysis was 29 lean and 15 participants with obesity. Data were analysed using mixed factorial ANOVA with ‘BMI status’ (lean/with obesity) as a between-subjects factor and ‘treatment’ (IN insulin/placebo) as a within-subjects factor. Composite VAS scores were created by averaging individual ratings according to the factor structure reported in [29]: ‘Arousal’ (alertness, drowsiness, happiness), ‘Appetite’ (hunger, fullness, desire to eat), ‘Negative Effects’ (disgust, anxiety, sadness, withdrawn), ‘Physical Effects’ (lightheaded, nausea, faint) and thirst and area under the curve was analyzed using the trapezoid method. Any effects that did not include the treatment factor of time, stimulus type and BMI status are not reported or followed-up. To test our a priori hypothesis that lean women and women with obesity might differ, we conducted planned *t* tests in the event of a significant main effect of insulin. Multiple comparisons were Holm-Bonferroni corrected. Violations of sphericity were addressed using the Greenhouse-Geisser correction.

## Results

### Demographics

Women with obesity had a higher BMI and self-reported more restrained and emotional eating in line with previous research (e.g. [38, 39]). See Supplementary Table 2 for participant demographics.

### Cookie intake

**Fig. 2:**
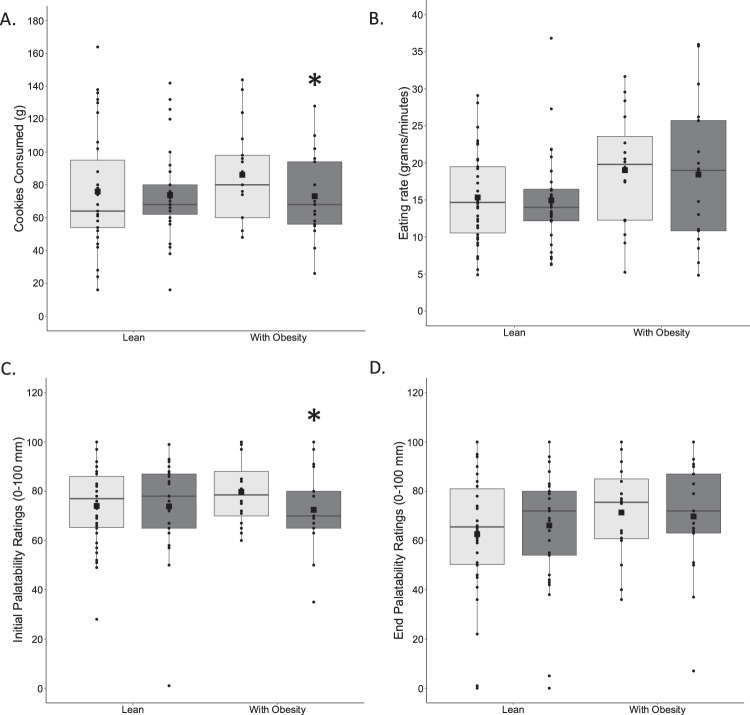
Eating-related measures. **A** depicts cookie intake of lean women and women with obesity in the placebo (light grey fill) and insulin conditions (dark grey fill). **B** depicts eating rate of lean women and women with obesity in the placebo and insulin conditions. **C** depicts palatability ratings of lean women and women with obesity at the beginning of the snack in the placebo and insulin conditions. **D** depicts palatability ratings of lean women and women with obesity at the end of the snack in the placebo and insulin conditions. Squares denote mean of each outcome. Asterisks denote follow-up significance at 0.05 level.

There was a main effect of IN insulin on cookie intake (*F*(1,50) = 4.59, *p* = 0.04, η_p_^2^ = 0.08) and follow-up tests revealed reduced intake of cookies for women with obesity (*t*(16) =−2.12, *p* = 0.05, *d* = 0.46) and no effect for lean women (*t*(34) =−0.55, *p* = 0.59, *d* = *0*.07). There was a main effect of IN insulin on initial ratings of cookie pleasantness (*F*(1,47) = 4.83, *p* = 0.03, η_p_^2^ = 0.09) and follow-up tests showed that IN insulin reduced initial cookie liking for women with obesity (*t*(15) =−2.87, *p* = 0.01, *d* = 0.42) but not for lean women (*t*(32) = −0.34, *p* = 0.74, *d* = 0.04). There was no main effect of IN insulin on rated pleasantness at the end of the session (F(1,47) = 0.01, *p* = 0.99, η_p_^2^ < 0.01). There was no effect of IN insulin on eating rate (*F*(1, 47) = 0.02, *p* = 0.89, η_p_^2^ < 0.01) (Fig. 2).

### Appetite and Mood

IN insulin reduced appetite (*F*(1,50) = 5.66, *p* = 0.02, η_p_^2^ = 0.10) and this effect was more pronounced for women with obesity (*t*(16) =−2.11, *p* = 0.051, *d* = 0.60) than for lean women (*t*(34) =−0.74, *p* = 0.47, *d* = 0.11). There were no significant main effects of IN insulin on arousal (*F*(1,50) = 2.16, *p* = 0.15, η_p_^2^ = 0.04), negative effects (*F*(1,50) = 1.39, *p* = 0.24, η_p_^2^ = 0.03), physical effects (*F*(1,50) = 0.06, *p* = 0.81, η_p_^2^ < 0.01), and thirst (*F*(1,50) = 0.01, *p* = 0.98, η_p_^2^ < 0.01).

**Fig. 3:**
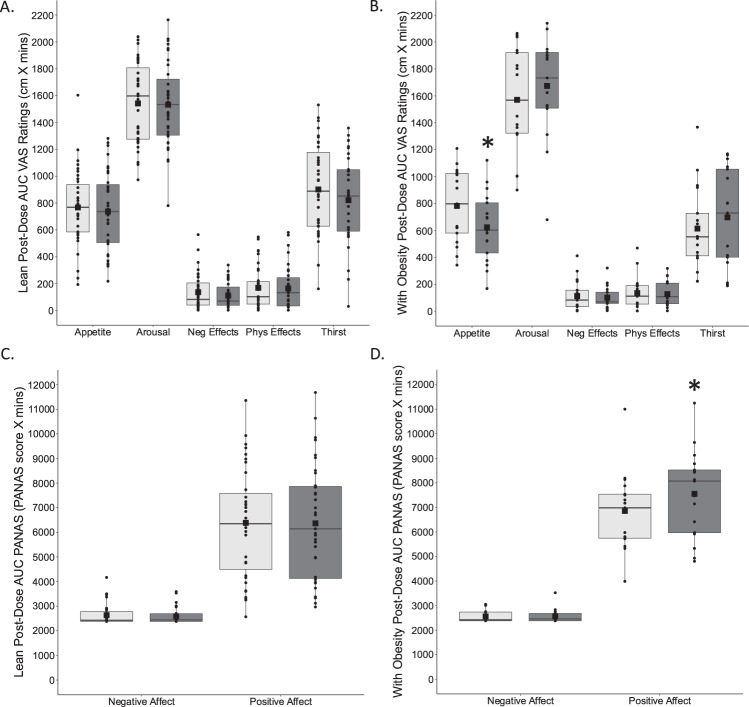
Appetite and mood. **A** depicts post-dose area under the curve (AUC) visual analogue scale (VAS) ratings of lean women between placebo (light grey fill) and insulin (dark grey fill) conditions. **B** depicts post-dose AUC VAS ratings of women with obesity in the placebo and insulin conditions. **C** depicts post-dose AUC Positive and Negative Affect (PANAS) ratings of lean women between placebo and insulin condition. **D** depicts post-dose AUC PANAS ratings of women with obesity in the placebo and insulin conditions. CM centimetre, Mins Minutes, Neg Effects negative effects, Phys Effects Physical Effects. Squares depict mean of each outcome. Asterisks denote follow-up significance at 0.05 level.

There was no significant main effect of IN insulin on PANAS ratings (*F*(1,47) = 3.78, *p* = 0.06, η_p_^2^ = 0.07), but the interaction between IN insulin and BMI was significant (*F*(1,47) = 5.47, *p* = 0.02, η_p_^2^ = 0.10) which was explained by a significant increase of positive affect (PA) ratings for women with obesity (*t*(16) = 2.86, *p* = 0.01, *d* = 0.42) (Fig. 3).

### Cognitive tasks

The only effect of IN insulin was improved accuracy for selection of self-referent positive adjectives and rejection of negative adjectives and slower RT on the n-back task for women with obesity. See below for statistical results and Supplementary Table 3 for descriptive statistics.

#### VPA

The effect of IN insulin condition on recall accuracy was not significant (*F*(1,50) = 0.01, *p* = 0.93, η_p_^2^ < 0.01).

#### ETB – ECAT

IN insulin improved accuracy for selection of positive adjectives for self-reference and rejecting negative adjectives for self-reference (*F*(1,49) = 6.76, *p* = 0.01, η_p_^2^ = 0.12). Follow-up tests revealed no significant differences of IN insulin on ECAT accuracy for lean women (*t*(32) = 1.87, *p* = 0.07, *d* = 0.22) nor women with obesity (*t*(16) = 1.72, *p* = 0.10, *d* = 0.49). The effect of IN insulin on RT (*F*(1,48) = 1.64, *p* = 0.21, η_p_^2^ = 0.03) was not significant.

#### ETB – EREC

Neither the effect of IN insulin on recall accuracy (*F*(1,50) = 0.67, *p* = 0.42, η_p_^2^ = 0.01) nor errors (*F*(1,50) = 0.15, *p* = 0.70, η_p_^2^ = 0.03) was significant.

#### ETB – EMEM

Neither the effect of IN insulin on accuracy (*F*(1,34) = 2.13, *p* = 0.15, η_p_^2^ = 0.06), commission errors (*F*(1,50) = 1.20, *p* = 0.28, η_p_^2^ = 0.02) nor RT (*F*(1,49) = 0.72, *p* = 0.40, η_p_^2^ = 0.02) was significant. The interaction between IN insulin and BMI for accuracy was significant (*F*(1,34) = 5.65, *p* = 0.02, η_p_^2^ = 0.14) but follow-up tests revealed no significant effects for either BMI group (*p*s > 0.05).

#### N-back

There was no effect of IN insulin on accuracy (*F*(1,27) = 1.08, *p* = 0.31, η_p_^2^ = 0.04), nor RT (*F*(1,45) = 1.89, *p* = 0.18, η_p_^2^ = 0.04). The interaction between IN insulin and BMI was significant for the RT measure (*F*(1,45) = 12.36, *p* < 0.01, η_p_^2^ = 0.22). Women with obesity were slower in the insulin condition than the placebo condition (*t*(15) = 3.33, *p* = 0.01, *d* = 0.56).

#### PRT recall

The effect of IN insulin on recall (*F*(1,49) = 0.01, *p* = 0.94, η_p_^2^ < 0.01) was not significant.

### Picture rating task

#### Pleasantness ratings

There was no significant effect of IN insulin on pleasantness ratings of food stimuli (*F*(1,43) = 1.32, *p* = 0.26, η_p_^2^ = 0.3).

#### fMRI

Statistically significantly greater (whole-brain FWE-corrected) blood-oxygen-level-dependent (BOLD) responses to food compared to non-food images (independent of condition) were observed in the left precuneus (*n voxels* = 427, *xyz* = [−6,51,18], Z = 5.46, *p* < 0.001), left superior frontal gyrus (*n voxels* = 611, *xyz* = [−6,63,−3], *Z* = 4.78, *p* < 0.001), left thalamus (*n voxels* = 99, *xy*z = [−3,−15,6], *Z* = 5.46, *p* = 0.031), left orbitofrontal cortex (*n voxel*s = 121, *xyz* = [−30,12,9], *Z* = 4.52,=0.016), and left inferior temporal cortex (*n voxels* = 99, *xyz* = [−30,66,45], *Z* = 4.16, *p* = 0.046). All *p*s FWE-corrected.

**Fig. 4:**
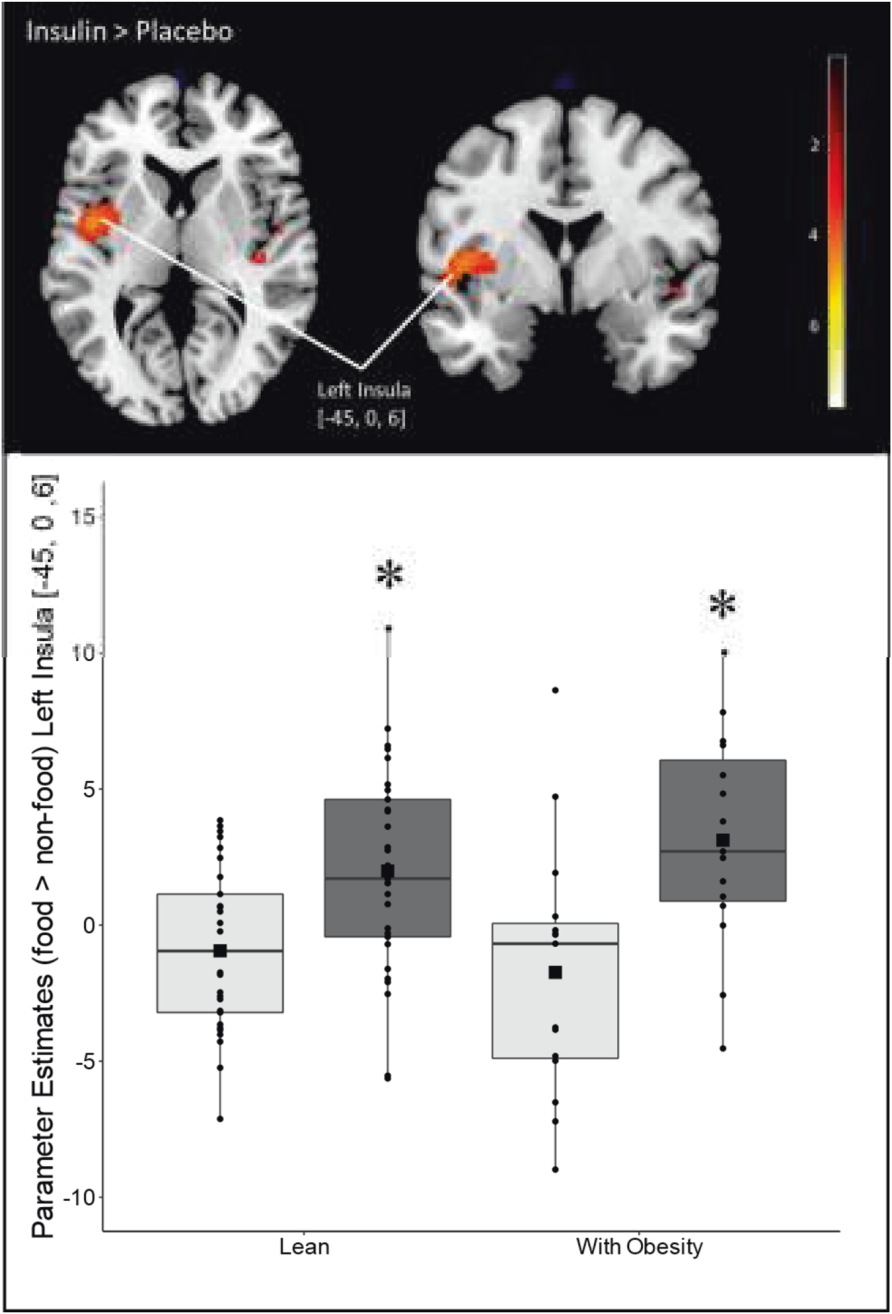
fMRI. Top panel: Significant cluster (FWE-corrected *p* < 0.05) in the left insula for contrast Insulin > Placebo. Figure thresholded at initial uncorrected detection threshold *p* < 0.001. Bottom panel: Parameter estimates from left insula cluster [−45,0,6]. Asterisks denote significant difference between placebo (light grey fill) and insulin (dark grey fill) for both lean participants and participants with obesity at 0.05 level. Squares depict the mean.

Under a whole-brain FWE-corrected significance threshold, the BOLD response in the left insula [−45,0,6] when viewing food versus non-food pictures was significantly greater in the insulin condition compared to placebo in both BMI groups (*n voxels* = 107, *Z* = 4.47, *p* = 0.038) (see Fig. 4). No regions showed an attenuation of activity after insulin compared to placebo. There was no interaction between food category and treatment.

### Blood insulin and glucose

Pre-dose blood glucose did not differ according to BMI (*F*(1,49) = 0.97, *p* = 0.33, η_p_^2^ = 0.02) or test day (*F*(1,49) = 0.32, *p* = 0.58, η_p_^2^ = 0.01) (Supplementary Table 4). IN insulin had no effect on blood glucose (*F*(1,48) = 0.10, *p* = 0.75, η_p_^2^ < 0.01) (Supplementary Table 4)

**Fig. 5:**
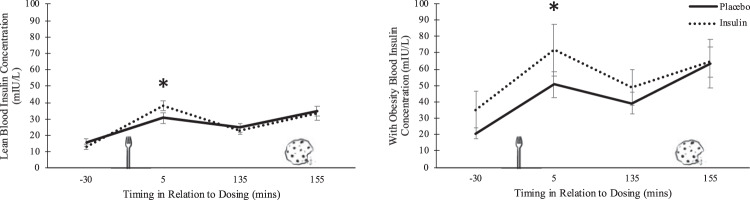
Blood insulin and glucose. Mean (±standard error of the mean) blood insulin concentration of lean women (left) and women with obesity (right). Fork image denotes timing of lunch and cookie image denotes timing of the snack. Asterisks denote follow-up significance at 0.05 level. Women with and without obesity had higher blood insulin concentrations 5 min post-dose in the insulin condition.

Women with obesity had higher baseline blood insulin than lean women (*F*(1,44) = 7.03, *p* = 0.01, η_p_^2^ = 0.14). Women with obesity had higher post-dose blood insulin concentrations than lean women (*F*(1,44) = 13.04, *p* < 0.01, η_p_^2^ = 0.23). There was a significant increase in blood insulin after IN insulin at 5 min post-dose (*t*(45) = 3.24, *p* < 0.01, *d* = 0.36) (see Fig. 5).

## Discussion

A post-prandial acute dose of IN insulin reduced cookie intake, appetite and the reward value of cookies in women. These effects were stronger for women with obesity compared with lean women. IN insulin improved mood in women with obesity. In both BMI groups, IN insulin increased the BOLD fMRI signal in the insula when viewing food (versus non-food) pictures. IN insulin did not improve cognitive function. Taken together these results suggest that IN insulin may reduce food intake by influencing food reward processes and that women with obesity may be more sensitive to these effects than lean women.

Given previous reports of blunted effects of IN insulin in individuals with obesity [13, 15, 18, 22, 40] presenting with peripheral insulin resistance [41] we hypothesized that participants with obesity could be less responsive to effects of IN insulin than lean women. In our sample, women with obesity had higher baseline blood levels of insulin compared with lean participants but did not differ in blood glucose levels and had normal HbA1c levels. This suggests that elevated insulin release may have compensated for reduced insulin sensitivity. It might be argued that IN insulin had effects in the women with obesity in our study because they retained sensitivity to intracerebral (rather than peripheral) insulin. In aged mice showing an attenuated effect on cortical activity of subcutaneously administered insulin, intracerebroventricular insulin delivery yielded a response similar to that seen in young animals [42]. Accordingly, the compromised brain effect of endogenous or peripherally administered insulin in obesity might stem primarily from attenuated insulin transport to the brain as a result of hyperinsulinaemia [43, 44], which can be overcome by directly targeting the brain with IN insulin [45].

The decrease in initial pleasantness of the palatable cookies after IN insulin suggests that the decrease in intake may be mediated by reduced food reward. Since the participants were in a post-prandial state when insulin was administered and cookies were offered, it is conceivable that IN insulin enhanced postprandial signals that reduce food reward when satiated. This is important because reductions in reward-related responding that are specific to the satiated state are likely to be effective in helping individuals to curb their appetite but are unlikely to reduce hedonic responding in general. However, in order to further understand the scope of the effect, future studies should examine the effects of IN insulin on intake of a range of foods varying in palatability and macronutrient content.

Further support for a specific effect of IN insulin on post-prandial signals comes from the fMRI data. Greater BOLD fMRI responses were elicited by viewing food compared to non-food images across several brain regions and in line with effects on eating behaviour, we observed an increased BOLD signal in response to food images in mid insula following IN insulin. This is consistent with evidence for a high density of insulin receptors in the insula [46] and that insulin facilitates neuronal firing in the insular cortex of mice [47] and increases neuronal excitability via a reduction in calcium dependent afterhyperpolarization [48]. The insula is hypothesized to integrate interoceptive and exteroceptive appetite and food reward signals [49, 50]. Hence, IN insulin-induced activation of the insula might enhance post-prandial signals that nutrients have been consumed, which in turn decreases the attractiveness of food.

Increased insula activation by IN insulin was observed in both BMI groups, but effects on appetite and food intake were observed only in women with obesity. Our task may have identified effects of IN insulin on processing of interoceptive signals in both lean women and women with obesity, but whether these actions were translated into reduced food intake could have depended upon downstream neural processes during food consumption that varied according to BMI status. One possibility is that women with obesity may have impaired interoception [51] and thereby benefit from enhanced interoceptive signals more than lean women.

Contrary to the results of Hallschmid et al. [16], we did not find a robust effect of IN insulin on cookie intake in lean women. Our study offered only one cookie type, while participants in the Hallschmid et al. [16] study were offered three varieties and IN insulin affected intake of the most palatable cookies. Variety stimulates intake [52] and so effects of IN insulin on cookie intake in lean women in the previous study [16] may have been related to the range of palatable options. There was substantial variability in the cookie intake of lean women in the placebo condition suggesting that not all found the cookies palatable in the present study. Therefore, it may be that because effects of IN insulin on intake are most pronounced for palatable foods we could have seen an effect if more palatable options had been provided.

Unlike previous studies [13–15] we saw no effects of IN insulin in brain regions associated with food reward or in the hypothalamus. However, most previous studies examined effects of IN insulin on resting state activity and in fasted participants [14, 15, 53], whereas we examined task related (picture rating) activity in satiated participants. This suggests that the effect of IN insulin on neural responses may vary depending upon nutritional state. Future investigations should examine how the neural responses to food cues in lean individuals and individuals with obesity are altered by IN insulin depending on their fed versus fasted state.

Intranasal insulin increased working memory RT of women with obesity but had no other effect on cognitive measures for either BMI group. This suggests that cognitive processes do not underlie the effects of IN insulin on food intake and appetite although the cognitive tasks used may have been insufficiently sensitive to detect effects of IN insulin on memory. Previous studies used a spatial memory task [4], which may be more sensitive to hippocampal effects of IN insulin.

We observed enhancement of mood after IN insulin for women with obesity. This is consistent with reports of IN insulin-induced mood improvement in men with obesity [9, 54]. The ability of IN insulin to decrease appetite and food intake in women with obesity combined with its mood enhancing effects are very promising in terms of therapeutic use of the hormone, especially given the co-morbidity of obesity, diabetes and mood disorders [55, 56]. Moreover, while there was an increase in blood insulin levels 5 mins after administration, suggesting some spillover of IN insulin into circulation, blood glucose levels were unchanged throughout the test day, highlighting the safety of the intervention.

This study has some limitations. The uneven and smaller sample size for participants with obesity may have masked statistical effects, although the large effect sizes suggest that they are robust.

In summary, we provide evidence that IN insulin reduces appetite and food intake and increases positive mood in women with obesity. These results show promise for the therapeutic use of IN insulin as a weight management option for women with obesity, particularly those with co-morbid mood disorders and further investigation of the longer-term effects of the hormone on weight in women with obesity is warranted.

## Supplementary information

Supplemental results

## Data Availability

Data for this study will be made available in a public archive following publication of this study. In the interim, data are available upon request
