# Supplementary material for: The effect of intranasal insulin on appetite and mood in women with and without obesity: an experimental medicine study
Source: Int J Obes (Lond). 2022 Apr 9;46(7):1319–27. doi: 10.1038/s41366-022-01115-1 (PMC9239904; doi:10.1038/s41366-022-01115-1)
Supplement: Supplementary file 1 — Supplemental results [file 41366_2022_1115_MOESM1_ESM.docx]

Supplementary Materials

*Table 1*. Inclusion/Exclusion Criteria

| Inclusion Criteria: | Exclusion Criteria: |
| --- | --- |
| 1. Aged 18-65 2. Female 3. Fluent English speaking 4. Body mass index (BMI) of 18.5-25 kg/m^2^ or > 30 kg/m^2^ 5. Medical clearance 6. Liking of study foods 7. No special diets (e.g., vegan or vegetarian) | 1. Weight loss of more than 5 kg in the 3 months prior to the test day 2. Diagnosed with an eating disorder 3. Metabolic disorder 4. Neurological disorder 5. Current mental health disorder determined via the SCID or self-reported diagnosis. 6. Substance use disorder 7. Current smoker 8. Pregnant or breastfeeding 9. Food allergies related to the study 10. MRI-related exclusion 11. Left-handed 12. Limited or increased perception of temperature changes 13. Pathological hearing 14. Surgical operation less than 3 months ago 15. Moderate or severe head injury 16. Acute illness or infection in the last month 17. Claustrophobia 18. Non-removable metal in the body |

*Table 2:* Participant Demographics

| Factor | Lean (n = 35) | With Obesity (n = 17) | *p*  value |
| --- | --- | --- | --- |
| Age (years) | 23.66 (4.80) | 26.00 (7.91) | 0.27 |
| BMI (kg/m^2^) | 22.21 (1.91) | 34.04 (3.38) | < 0.01 |
| HbA1c (mmol/mol) | 33.03 (3.16) | 34.21 (3.98) | 0.09 |
| BDI (Max = 63) | 2.63 (3.62) | 5.18 (5.43) | 0.09 |
| PFS (Max = 75) | 36.51 (12.75) | 40.35 (11.82) | 0.30 |
| DEBQ (Max = 5) |  |  |  |
| *Restraint* | 2.31 (0.49) | 2.82 (0.38) | < 0.01 |
| *External* | 3.04 (0.50) | 3.16 (0.33) | 0.32 |
| *Emotional* | 2.06 (0.66) | 2.65 (0.67) | = 0.01 |

*Table 2.* Data are expressed as mean and ± standard deviation. Questionnaire measures are included with the maximum possible score. BMI: Body Mass Index. HbA1c: glycated haemoglobin. BDI: Beck Depression Inventory – II. PFS: Power of Food Scale Total (aggregate of Food Available, Food Present, and Food Tasted factors). DEBQ: Dutch Eating Behaviour Questionnaire.

**Table 3: Cognitive Task Results**

| Lean | | | | | With Obesity | | | | |
| --- | --- | --- | --- | --- | --- | --- | --- | --- | --- |
|  | **Placebo** | | **Insulin** | | | **Placebo** | | **Insulin** | |
| Task | Accuracy | RT | Accuracy | RT | | Accuracy | RT | Accuracy | RT |
| VPA (%) correct |  |  |  |  | |  |  |  |  |
| *Immediate* | 25.57(9.47) | -- | 25.97(11.25) | -- | | 27.65(9.31) | -- | 27.12(10.19) | -- |
| *Delayed* | 35.40(10.93) | -- | 37.11(11.34) | -- | | 40.59(8.85) | -- | 39.47(9.74) | -- |
| ECAT (% correct) |  |  |  |  | |  |  |  |  |
| *Positive* | 90.00(9.03) | 772.62(166.34) | 92.06(6.86) | 783.56(179.51) | | 91.17(5.52) | 721.19(123.34) | 92.55(6.30) | 769.00(168.42) |
| *Negative* | 91.37(6.88) | 803.44(185.96) | 92.26(7.47) | 833.47(162.21) | | 88.81(7.63) | 778.38(132.89) | 92.16(5.40) | 808.25(150.95) |
| EMEM (% correct) |  |  |  |  | |  |  |  |  |
| *Positive* | 85.21(8.46) | 1046.44(234.86) | 87.39(8.53) | 1111.38(274.66) | | 85.36(9.49) | 1128.00(157.93) | 81.54(9.39) | 1135.53(308.08) |
| *Negative* | 75.80(10.27) | 1187.44(269.06) | 75.95(10.54) | 1239.77(325.47) | | 76.93(13.93) | 1250.76(233.74) | 71.03(8.21) | 1248.18(310.97) |
| EREC (items) |  |  |  |  | |  |  |  |  |
| *Positive* | 5.20(2.39) | -- | 5.03(2.67) | -- | | 5.30(3.12) | -- | 4.71(2.64) | -- |
| *Negative* | 5.17(2.8) | -- | 5.37(2.71) | -- | | 5.47(2.62) | -- | 4.76(1.68) | -- |
| N-back (% correct) |  |  |  |  | |  |  |  |  |
| *2-back* | 77.42(11.82) | 888.16(215.99) | 82.16(12.43) | 827.32(212.65) | | 83.70(11.64) | 802.41(227.45) | 83.50(13.35) | 916.22(182.88) |
| *3-back* | 74.84(11.41) | 860.94(247.90) | 76.32(12.61) | 828.71(246.12) | | 79.60(16.93) | 759.94(190.73) | 83.10(13.27) | 858.99(210.59) |
| PRT (% total possible) |  |  |  |  | |  |  |  |  |
| *Food* | 17.39(6.00) | -- | 17.79(6.94) | -- | | 19.04(6.15) | -- | 17.87(5.63) | -- |
| *Object* | 10.37(3.57) | -- | 11.07(4.04) | -- | | 9.93(4.93) | -- | 10.22(3.65) | -- |
| Emotional Test Battery Commission Errors | | | | | | | | | |
| EREC (items) |  |  |  |  | |  |  |  |  |
| *Positive* | 3.46(2.05) | -- | 4.14(3.22) | -- | | 4.18(2.19) | -- | 4.24(2.49) | -- |
| *Negative* | 1.89(1.79) | -- | 1.43(1.61) | -- | | 1.71(1.53) | -- | 1.76(1.56) | -- |
| EMEM (% incorrect) |  |  |  |  | |  |  |  |  |
| *Positive* | 34.29(18.99) | -- | 33.72(18.93) | -- | | 42.34(22.27) | -- | 40.40(21.29) | -- |
| *Negative* | 20.47(12.97) | -- | 20.19(13.68) | -- | | 23.73(9.86) | -- | 18.24(11.45) | -- |
| Delay Discounting Results (AUC) | | | | | | | | | |
| Money | 77.95(16.89) | -- | 77.70(18.26) | -- | | 74.32(19.99) | -- | 73.79(15.11) | -- |
| Food | 58.46(34.85) | -- | 57.94(29.67) | -- | | 55.37(31.85) | -- | 63.39(30.56) | -- |

*Table 3*. Data presented as mean *±* standard deviation. RT: reaction time, ECAT: Emotional Categorisation Task – positive and negative valence, EREC: Emotional Recall Task – positive and negative valence, ECAT: Emotional Recognition Memory Task – positive and negative valence, PRT: Picture Rating Task recall. EREC data expressed as items remembered or falsely remembered. Delay discounting data expressed as area under the curve. PRT results expressed as percentage correct out of total possible. All other results expressed as percentage correct or incorrect. The main effect of IN insulin condition on ECAT accuracy was statistically significant (*p* < 0.05).

**Table 4: Blood Glucose Results**

|  | Lean  _______________________ | | With Obesity  _______________________ | |  | | |
| --- | --- | --- | --- | --- | --- | --- | --- |
| Draw | **Placebo** | **Insulin** | **Placebo** | **Insulin** | **Analysis factors** | **Pre-dose result** | **Post-dose result** |
| -30 minutes | 4.72 (0.61) | 4.54 (0.50) | 4.69 (0.41) | 4.79 (0.67) | IN insulin | (*F*(1, 49) = 0.32, *p* = .58, η_p_^2^ = .01) | (*F*(1, 48) = 0.10, *p* = .75, η_p_^2^ < .01) |
| 5 minutes | 4.65 (0.71) | 4.59 (0.80) | 4.74 (0.53) | 4.77 (0.77) | IN insulin * BMI | (*F*(1, 49) = 1.63, *p* = .21, η_p_^2^ = .03x) | (*F*(1, 48) = 0.06, *p* = .81, η_p_^2^ < .01) |
| 135 minutes | 5.01 (0.65) | 4.84 (0.62) | 4.91(0.73) | 4.74 (0.51) | IN insulin * time |  | (*F*(3, 48) = 1.67, *p* = .18, η_p_^2^ = .03) |
| 155 minutes | 4.74 (1.03) | 5.02 (0.74) | 5.07 (0.64) | 5.09 (0.55) | IN insulin * BMI * time |  | (*F*(3, 48) = 0.29, *p* = .80, η_p_^2^ = .01) |
| 300 minutes | 5.32 (0.65) | 5.47 (0.71) | 5.09 (0.72) | 5.35 (0.62) |  |  |  |

*Table 4*: Mean and ± standard deviation presented for glucose concentration (mmol/L) values. Dashed line indicates timing of the snack in relation to the blood draws. The main effect of IN insulin condition, and the interaction between IN insulin condition, BMI status, and blood draw were not statistically significant.
